# Effects on incident reporting after educating residents in patient safety: a controlled study

**DOI:** 10.1186/1472-6963-11-335

**Published:** 2011-12-12

**Authors:** José D Jansma, Cordula Wagner, Reinier W ten Kate, Arnold B Bijnen

**Affiliations:** 1 Foreest Medical School, Medical Center Alkmaar, Wilhelminalaan 12, 1815JD Alkmaar, the Netherlands; 2 EMGO Institute for Health and Care Research, VU University Medical Center, Van der Boechorststraat 7, 1081BT Amsterdam, the Netherlands; 3 NIVEL Netherlands Institute for Health Services Research, Otterstraat 118 - 124, 3500BN Utrecht, the Netherlands; 4 Kennemer Gasthuis, Boerhaavelaan 22, 2035 RC Haarlem, the Netherlands; 5 VU University Medical Center, Institute for Education and Training, Van der Boechorststraat 7, 1081BT Amsterdam, the Netherlands

## Abstract

**Background:**

Medical residents are key figures in delivering health care and an important target group for patient safety education. Reporting incidents is an important patient safety domain, as awareness of vulnerabilities could be a starting point for improvements. This study examined effects of patient safety education for residents on knowledge, skills, attitudes, intentions and behavior concerning incident reporting.

**Methods:**

A controlled study with follow-up measurements was conducted. In 2007 and 2008 two patient safety courses for residents were organized. Residents from a comparable hospital acted as external controls. Data were collected in three ways: 1] questionnaires distributed before, immediately after and three months after the course, 2] incident reporting cards filled out by course participants during the course, and 3] residents' reporting data gathered from hospital incident reporting systems.

**Results:**

Forty-four residents attended the course and 32 were external controls. Positive changes in knowledge, skills and attitudes were found after the course. Residents' intentions to report incidents were positive at all measurements. Participants filled out 165 incident reporting cards, demonstrating the skills to notice incidents. Residents who had reported incidents before, reported more incidents after the course. However, the number of residents reporting incidents did not increase. An increase in reported incidents was registered by the reporting system of the intervention hospital.

**Conclusions:**

Patient safety education can have immediate and long-term positive effects on knowledge, skills and attitudes, and modestly influence the reporting behavior of residents.

## Background

Ten years ago, the gravity of the problems threatening patient safety became more visible and the need for patient safety education became adopted in policy plans worldwide [1-3]. Patient safety education focuses on the acquisition of knowledge, attitudes and skills to support changes in behavior in order to deliver safer care [4]. A major part of the patient safety principles involve non-technical skills and therefore are not necessarily discipline-specific [5,6]. An important patient safety related topic is the voluntary and non-punitive reporting of unintended or unexpected events which might or did lead to harm for one or more patients. This can be a valuable method both to gain insight into the occurrence and causes of incidents and to identify risk factors which should be acted upon to improve patient safety [7-9]. Systems for reporting incidents in other high-risk sectors, such as the aviation and the petrochemical industry, have demonstrated to be useful as they resulted in measurably safer systems [9]. There are three principal conditions for creating an effective reporting system: 1] health care workers must be aware of the importance of reporting incidents (attitudes), 2] they need to know how to report an incident (knowledge), and 3] they must be able to recognize risky situations (skills) [10]. Patient safety education is perceived as a successful method to achieve these principal conditions and to stimulate an active reporting culture [6].

Medical residents are key figures in the care process and for several reasons it is expected that patient safety education for this group in particular can lead to valuable results. Firstly, residents are considered to be a group which can contribute to long-lasting benefits, as these physicians are at the beginning of their career and they are the medical specialists of the future. Secondly, residents provide much of the direct patient care [11]. Thirdly, they are considered a fragile link in the care process as a lack of work experience and a high work-pressure among residents increases hazardous situations [12,13]. Moreover, research showed that medical trainees' knowledge of patient safety across a broad range of training levels, degrees and specialties was limited [14], and that physicians in general have a relatively low rate of incident reporting [15].

Although more attention is being paid to patient safety in medical education, only a few studies assessed the effects of patient safety education for residents on their incident reporting behavior [12,16-18]. None of these studies had a controlled design and none described measuring changes in actual behavior as derived from existing (e.g. hospital wide) reporting systems. The two studies that did use objective outcome measures both did this by distributing a specific study-related reporting tool (i.e. "the safety journal" [12] or "the outcomes card" [17]), which was demonstrated to be suitable to show skills in incident reporting and analysis. These studies also demonstrated that residents were able and willing to report and analyze incidents using these tools. Two other studies merely used subjective outcome measures, focusing on residents' self-assessed attitudes, intentions and behavior concerning incident reporting [16,18]. These studies both found a discrepancy between residents' reporting intentions and behavior.

We wanted to gain more insight into residents' incident reporting behavior by combining objective and subjective outcome measures, in a controlled design where possible. We assessed the immediate and longer-term effects of a patient safety course for residents by inclusion of data from hospital-wide reporting systems, a specific study-related reporting tool and residents' self-assessments. In doing so, we focused on reporting behavior and on the four antecedents of behavior that we hoped to affect by educating residents: knowledge, skills, attitudes and intentions.

## Methods

### Setting & sample

This study involved two comparable large general teaching hospitals located in the Netherlands, which have a similar web-based system for confidential and voluntary reporting of incidents. One hospital was used as the intervention hospital and the other one as the control hospital. In total, about 130 residents were working in the intervention hospital and about 90 residents were working in the control hospital at the start of the study. Residents of the intervention hospital were obliged to attend the patient safety course if their contract within the hospital continued for at least three months after the course ended. However for some residents it was not possible to attend, for example because of scheduled holidays, or maternity leave. If a resident was not able to attend the first course, we tried to let him/her attend the second course.

Controls were only recruited from disciplines comparable to those of the course participants and if their contract within the hospital continued for at least three months after the course ended. The national rules and regulations regarding health services research were followed. The Scientific Research Review Board of the VU University Medical Center provided a waiver for this study.

### Patient safety course

We organized two separate patient safety courses for residents from multiple specialties of the intervention hospital, both starting in September 2007. Both courses were identical in content and aimed at increasing knowledge of patient safety and skills to recognize and cope with unintended events and unsafe situations in an early stage. Each course spanned a period of three months and consisted of one plenary day, followed by two half-days in smaller groups. We used a mixture of educational methods to create an interactive learning environment.

**Figure 1:**
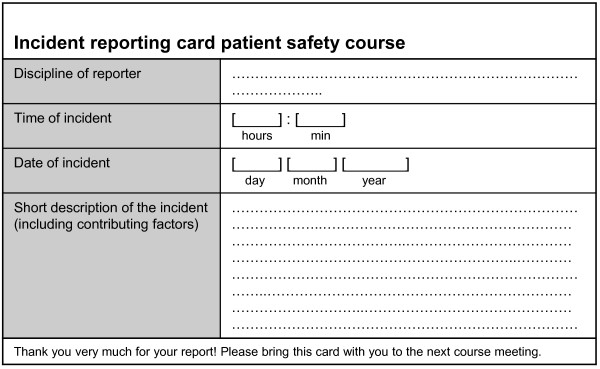
**Incident reporting card**.

The importance of incident reporting was stressed throughout the course. During the first course meeting participants practiced incident analysis by performing root cause analyses with the Prevention and Recovery Information System for Monitoring and Analysis (PRISMA medical version) [19]. This method for analyzing incidents distinguishes four main categories of causes: technical, organizational, human and patient related. To get used to noticing and reporting incidents and to become aware of the importance of reporting, each participant received ten pocket-size reporting cards at the first course meeting (Figure 1). Filling out these cards by health care workers had proved to be useful in previous research [20]. We asked residents to fill out the cards during their clinical work and to analyze five incidents by investigating the underlying causes. Development and content of the course were described in more detail elsewhere [21].

### Data collection

We collected data in three different ways.

1] We distributed questionnaires before, directly after and three months after the course was given (M1-M3). These measured self-assessed changes in *knowledge, skills, attitudes, intentions *and *behavior *of respondents. The post-course measurement of the first course took place at about the same time (+/- two weeks) as the pre-course measurement of the second course, allowing for an internal cross-over comparison. At the same points in time we sent questionnaires to the external controls (hereafter referred to as "control group"). We sent follow-up measurements only to those controls who responded to the first measurement. To stimulate responsiveness, we raffled lottery tickets among the control group. We did not raffle lottery tickets among the intervention groups. All questionnaires were encoded and processed confidentially.

2] We gathered and processed incident reporting cards and root cause analyses that were filled out by course participants between the first and second course meeting. This provided additional insight into the *skills *and behavior of residents to notice incidents.

3] The hospital-wide digital incident reporting systems of both hospitals provided information about the factual reporting *behavior *of residents.

### Questionnaire

We developed a 17-item questionnaire (see Additional File 1). At each measurement we asked the residents about a selection of these items. We put the last three items only to the intervention groups, all other items were presented to the controls as well. The first two items referred to the experienced level of *knowledge *of the respondents concerning handling incidents and patient safety improvement in general, which was measured on a 4-point Likert scale (Strongly disagree/Disagree/Agree/Strongly agree). For item 3-11, three answer options were given (No/Cannot decide/Yes). Item 12 was open ended. Item 3-5 assessed course participants' changes in *skills *with regard to noticing and analyzing incidents. Item 6-11 consisted of vignettes that provided insight into residents' *attitudes *towards incident reporting in specific situations. Incident reporting in this setting meant voluntary reporting of incidents by filling out a digital registration form at the hospital incident reporting system. As the patient safety course stressed the importance of making a report of all incidents, it would have been correct to consider all the vignettes in the questionnaire worth a report. Item 12-17 focused on incident reporting *attitudes, intentions *and *behavior *and were based on a questionnaire of Coyle *et al. *(2005), who measured the impact of an educational program for graduate trainees as well. Item 1-11 were developed by content experts (ABB & CW) and based on their experience in health care and patient safety research. An overview of the different data collection methods that we used in this study is presented in table 1.

**Table 1:** Overview of different data collection methods

	Objective outcome	Subjective outcome (questionnaire)	Intervention groups	Control group
Knowledge		X	x	x
Skills	x (number of reporting cards)	X	x	
Attitudes		x	x	x
Intentions		x	x	x
Behavior	x (hospital reporting system)	x	x	x

### Analysis

We processed data in SPSS and used a probability of *p *≤ 0.05 (two-tailed) to establish statistical significance. We assessed comparability of residents' characteristics for nominal measures by Chi-square analysis, and for scale measures by the Independent-Samples T-test and the Mann-Whitney test. We analyzed questionnaires with Friedman's test for analysis of three related assessments. We used the Paired Samples T- test and the Wilcoxon Signed Ranks Test to analyze two related measurements. To be able to compare the questionnaire's items, we transformed the 4-point Likert scale variable into a 0-2 scale (0 = Strongly disagree; 2 = Strongly agree). We created a sum variable to analyze the six vignette items. The Mann-Whitney test was used for comparing answers between intervention groups and control group. We used logistic regression (enter and stepwise) to determine the relation between the items measuring incident reporting intentions or behavior and the items measuring knowledge, skills, attitudes or intentions. To do this, we first transformed the items measuring intentions and behavior into a binary variable by merging the answer categories 'No' and 'Cannot decide'. Data from the hospital reporting system were analyzed using the Chi-square test.

## Results

### Respondents

In total, 44 (34%) residents were eligible to attend and attended one of the two patient safety courses. Forty-three percent (n = 19) of these residents attended the first course, 57% (n = 25) participated in the second course. The response rates among these course participants were: M1: 100% (n = 44), M2: 98% (n = 43) and M3: 100% (n = 44). At the control hospital we approached 57 (63%) residents at the first measurement. The response rates of the control group were: M1: 63% (n = 32), M2: 73% (n = 22) and M3: 96% (n = 23). Eight controls left the hospital during the study period and therefore had to be excluded from some of the measures. Table 2 shows the respondents' characteristics.

**Table 2:** Characteristics of respondents

Characteristics	Intervention (n = 44)	Control (n = 32)
Age, years		
Range	25.2-54.0	24.2-50.4
Mean	32.2	29.6
Sex, n (%)		
Male	15 (34)	12 (38)
Female	29 (66)	20 (63)
Discipline, n (%)		
Surgical	20 (45)	11 (34)
Non-surgical	24 (55)	21 (66)
Time at institution, years		
Range	0.18-10.7	0.03-11.7
Mean	1.35	1.36
At baseline measurement declared to have reported an incident within last six months	18 (41)	11 (52)

### Comparability of groups

Analyses of respondents' characteristics demonstrated that the intervention groups and the control group were comparable and formed representative samples of the hospitals' resident population. Questionnaires' outcomes at the first measurement did not show differences between the two intervention groups, nor between the control group and the intervention groups. Hospital incident reporting systems showed no significant differences in residents' reporting behavior during a period of eight months before the first course started.

### Patient safety knowledge (table 3)

At baseline, 60% (n = 26) of the course participants agreed or strongly agreed that they knew what to do if they were involved in an incident, and 48% (n = 20) agreed or strongly agreed to have sufficient knowledge to improve patient safety at the department. After the course they experienced a significant positive change in their level of knowledge. Post-course, 86% (n = 36) agreed or strongly agreed that they knew what to do if they were involved in an incident, and 88% (n = 36) agreed or strongly agreed to have sufficient knowledge to improve patient safety at their department. The control group demonstrated no significant changes in knowledge.

**Table 3:** Course participants' answers to knowledge, skills, attitudes, intentions and behavior items, mean (SD)*

	Pre-course^†^	Post-course^†^	Follow-up^†^	Significance
1. I have the feeling that currently I know what to do in case I will be involved in an incident.	1.10 (0.43)	1.35 (0.43)		*p *= 0.003^‡^
2. I have the feeling that currently I am having sufficient knowledge to improve patient safety at my department.	1.06 (0.47)	1.30 (0.30)		*p *= 0.019^‡^
3. Because of the course I am more able to signal unsafe situations.			1.55 (0.82)	^§^
4. Because of the course I can recognize that multiple factors contribute to an incident.			1.85 (0.53)	^§^
5. During the course I learned how to analyze incidents systematically.			1.80 (0.61)	^§^
*Do you consider the following events worth a report?*				
6. You bring the wrong patient to the operating room, you notice your mistake in time and pick up the right person.	0.82 (0.92)	1.07 (0.99)	1.27 (0.87)	*p *= 0.009^¶^
7. At the start of your shift you notice that Mr. B's heparin pump is adjusted too high.	1.68 (0.64)	1.74 (0.58)	1.75 (0.58)	*p *> 0.05^¶^
8. You requested with high speed the results of a laboratorial test but you received them much too late.	1.23 (0.86)	1.47 (0.77)	1.36 (0.78)	*p *> 0.05^¶^
9. The treatment policy of Mrs. X changed, but so far there is no notification of this in her status.	1.07 (0.87)	1.56 (0.77)	1.39 (0.78)	*p *< 0.001^¶^
10. You notice that the ampoules are not placed as usual, you were not informed about a change in policy.	0.68 (0.83)	1.02 (0.94)	1.02 (0.87)	*p *> 0.05^¶^
11. On hindsight it became clear that the diagnosis of Mr. M was wrong, the patient did not experience any disadvantages.	0.55 (0.73)	1.05 (0.90)	0.84 (0.83)	*p *= 0.004^¶^
12. Do you think it is important for residents to report medical incidents *without *harm for the patient(s)?	1.86 (0.35)	1.88 (0.39)	1.91 (0.36)	*p *> 0.05^¶^
13. Do you think it is important for residents to report medical incidents *with *harm for the patient(s)?	1.98 (0.15)	1.95 (0.21)	1.98 (0.15)	*p *> 0.05^¶^
14. Are you seriously considering reporting medical incidents within the next six months?	1.84 (0.43)	1.88 (0.39)	1.82 (0.95)	*p *> 0.05^¶^
15. Are you planning to start reporting within the next month?^#^	1.43 (0.84)	1.66 (0.80)	1.52 (0.79)	*p *> 0.05^¶^
16. Have you reported a medical incident within the last six months?	0.88 (0.99)	0.93 (0.99)	0.95 (1.01)	*p *> 0.05^¶^
17. If you have, how many incidents did you report within the last six months?**	1-14	1-6	1-7	*p *=
	2-1	2-9	2-6	0.002^¶^
		4-1	3-3	
		5-2	4-3	
		7-1	5-1	

*Not all residents who responded to the questionnaire filled out all items. The response rate per item varied between 40 (91%) and 44 (100%).

†Item 1&2: 0 = Strongly disagree; 2 = Strongly agree. Item 3-16: 0 = No; 2 = Yes.

‡Measured using the Wilcoxon Signed Ranks Test.

§Not applicable.

¶Measured over all three measurements at once using the Friedman Test.

#Answers included only those respondents who, at the first measurement, declared not to have reported an incident within the last six months (item 11).

**Number of incidents - number of residents.

### Skills of course participants (table 3 &4)

Three months after the course, almost three-quarters of the participants declared that the course had contributed to an improved signaling of unsafe situations. More than 90% of the participants acknowledged that because of the course they were able to recognize that multiple factors contribute to incidents and that the course had taught them how to analyze incidents systematically. In total, 165 incident reporting cards were filled out by 36 (82%) course participants (mean: 4) and 31 root cause analyses were performed by 14 (32%) of these residents (mean: 2), which demonstrated the ability of the residents to notice and analyze incidents. A selection of reported incidents, in which the variety in types and causes of the identified incidents is illustrated, is shown in table 4.

**Table 4:** Selection of reported incidents

Discipline	Description of incident	Causes mentioned
Internal medicine	Unnecessarily high glucose level.	- Notification by supporting personnel was too late (attending resident was supposed to be called by telephone about this) - Very busy at department
Multiple	Preventable infections.	- Health care workers do not always wash their hands before touching another patient - Laziness - Time pressure - Unaware of seriousness of the consequences
Gynecology	Delayed delivery of a child in foetal need.	- Suction pump out of order (probably caused by bump to door pillar) - Health care worker's ignorance of slurp sounds made by suction pump - Insufficient checking of the suction pump
Revalidation	Needle (with cover) found in bed with patient.	- Incompetent laboratory assistant - Patient also had not noticed the needle - Very busy at department
Emergency medicine	Patient needed plaster bandage, but was sent home without.	- Miscommunication between physician and nurse - Nurse followed own policy
Pediatrics	Patient needed isolated room, but was admitted to a room with multiple beds. The other beds in the room were kept empty.	- There were no isolated rooms available - It was late in the evening
Anesthesia	Unknown amount of local anesthetic was given to patient during spinal anesthesia.	- Hastiness - Connection between spinal needle and sprayer was insufficient and fell apart
Orthopedics	Decubitus ulcer.	- Decubitus prevention plan not followed by nurses - Busy nightshift
General surgery	Patient was kept sober all day and was prepared for the operation room, but the operation was not performed that day.	- Operation was not registered on operating list - Unclear description in patient's chart - Unclear treatment policy - Order was not checked - Miscommunication between health care workers

### Incident reporting attitudes (figure 2 & table 3)

At the baseline measurement a minority of residents considered the vignettes worth reporting and a greater part of residents judged reporting by residents to be important for incidents *with *harm, as well as for incidents *without *harm. Analysis of vignettes at all three measurements showed significant positive changes (*p *< 0.001) in the intervention groups. Immediately after the course (*p *< 0.001), as well as when pre-course measurements were compared to follow-up measurements (*p *= 0.002), residents more often considered the incidents proposed in the questionnaire worth reporting. After participating in the course, more residents judged that reporting incidents *without *harm is necessary, but this change did not reach statistical significance. All but one or two residents thought it important to report incidents *with *harm for the patient. The control group showed no significant lasting changes in attitudes.

**Figure 2:**
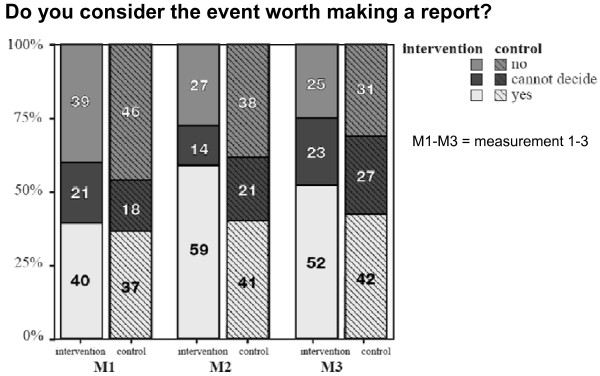
**Respondents' answers to six vignette items, %**.

### Incident reporting intentions (table 3)

At baseline intentions to report incidents within the next six months and intentions to start reporting within the next month were present among the majority of the respondents. These intentions remained stable immediately after the course as well as during follow-up. The controls did not demonstrate significant changes over time, either. None of the independent variables (knowledge, skills or attitudes) made a significant contribution to the incident reporting intentions.

### Incident reporting behavior (table 3)

At the baseline measurement less than half of the residents declared to have reported an incident within the last six months. According to the answers to the questionnaire the number of reported incidents increased significantly after the course. No significant changes were found in the number of residents who reported incidents. The control group did not show any changes over time in the number of reporters or the number of reports. At the intervention hospital the reporting system registered an increase of 130% (from 19 to 44 reports, *p *= 0.001. Mean per resident: from 0.15 to 0.32 reports) of reported incidents by residents eight months after the first course had started compared to eight months prior to the first course meeting. This increase remained stable over another period of eight months. Increased reporting was not restricted to the course participants: other residents from the intervention hospital also reported more incidents. In the same period the incident reporting system of the control hospital registered a reduction in reports by residents (from 10 to 7 to 0, *p *= 0.003). None of the independent variables (knowledge, skills, attitudes or intentions) made a significant contribution to the incident reporting behavior.

## Discussion

Reporting of incidents is an important step towards improving the safety of patients. The patient safety course for residents that we evaluated in this controlled study repeatedly highlighted the importance of reporting incidents and offered opportunities to practice noticing and analyzing incidents. We expected to measure improvements in residents' incident reporting knowledge, skills, attitudes, intentions and behavior.

Five major outcomes can be derived from our study results. 1] After attending the course residents declared to have more knowledge about handling incidents and patient safety improvement in general. 2] Course participants agreed that the course had contributed to the acquisition of skills for noticing and analyzing incidents. The reporting cards that were filled out during the course confirmed the presence of these skills. 3] After attending the patient safety course, the residents' ability to assess what kind of events are worth reporting improved significantly and consistently. 4] Residents are aware of the importance of reporting incidents and most of them have intentions to report. 5] The course seemed to have a positive effect on the incident reporting rate of attendants that had reported incidents previously, but the course did not seem to contribute to the activation of attendants' incident reporting behavior. Thus, although residents' knowledge, skills, attitudes and intentions to report incidents appeared to be present, these did not turn out to be an unambiguous predictor for the reporting behavior of residents.

While an increase of reported incidents by residents was registered by the incident reporting system of the intervention hospital, the number of these reports still remained low. However, at both hospitals the number of incidents that residents declared to have reported was higher than the hospital reporting system indicated. An explanation for this might be that residents who notice incidents are requesting other health care workers (e.g. nurses) to make the report, in which case it is possible that residents are not traced by the hospital reporting system, though residents themselves might judge this as having reported an incident.

Previous studies also found positive changes in attitudes after patient safety education for residents, and a discrepancy between intentions to report incidents and factual changes in reporting behaviors [16,18]. However these studies did not include an assessment of residents' reporting skills. Our study showed that a patient safety course together with an incident reporting tool, can result in a large number of incidents observed by residents, which demonstrates the presence of reporting skills, and at the same time can improve residents' knowledge, attitudes and modestly their behavior. Some of the items, in particular those that measured residents' attitudes and intentions, were already very positive at baseline, which made it very difficult to measure significant improvements over time. Compared to pre-course data from an earlier study [18], the attitudes and intentions concerning incident reporting measured in our present study were more positive at baseline. This difference could be the result of an increased attention to patient safety worldwide over the last few years. Since January 2008 the Dutch government obligates all Dutch hospitals to introduce safety management systems [3]. Decentralized incident reporting is one of the key elements of these systems. The registered increase in the incident reporting system of the intervention hospital may have been initiated by an increased attention to patient safety in the intervention hospital, which occurred concurrently with the organiza-tion of the patient safety courses. Besides, it is likely that the trained residents indirectly contributed to the increase in reports by their colleagues, as the trained residents were working closely together with non-trained residents, and no increase occurred at the control hospital. Patient safety education for medical professionals in combination with other patient safety projects also resulted in long lasting benefits at hospitals elsewhere [22].

### Incident reporting barriers

Although the number of reported incidents by residents in the hospital reporting system remained low, this probably was not caused by an absence of incidents in their work, as research revealed that residents are regularly involved in incidents [23-30] and residents filled out a large number of incident reporting cards during the course. The non-occurrence of actual incident reporting behavior might be explained by the Theory of Planned Behavior (TPB) [31]. This widely used social-psychological model distinguishes the following four aspects that influence behavior: attitudes, subjective norms, behavioral control and intentions. We found that the intentions and the attitudes of our residents concerning incident reporting were mainly positive. Therefore, it is likely that the incident reporting barriers that our residents experienced were related to the other two aspects of the TPB: the subjective norms and the behavioral control. In this study, we did not make an inventory of possible barriers that might discourage incident reporting among the residents. Nevertheless, other publications [15,16,32] suggested several barriers, related to human as well as system factors, that could hinder incident reporting. For example, a lack of encouragement by faculty, a lack of timely and high-quality feedback on medical incident reports, and fear of compromising one's career or personal reputation might contribute to discouraging subjective norms. A low perceived behavioral control might be associated with time constraints, complex reporting systems and forgetfulness. Relating identified barriers to the aspects of the TPB can be useful as a guidance for the development and evaluation of interventions to bring on behavioral changes [33].

### Limitations

Although the hospitals in this study can be considered comparable and general, one must be aware that a limited number of residents, of only one institution was trained, and only one other institution acted as control. Therefore the ability to generalize the outcomes for other settings may be restricted. Moreover, part of the results are based on the perception of the respondents, which might provoke social desirability bias. We tried to reduce this limitation by including objective data from the hospital digital reporting system and the specifically study-related reporting tool. The questionnaire that we used was not validated, but a major part of it had been used in other studies previously. We cannot be certain that the results are caused entirely by the course, as residents are continuously exposed to a diversity of stimuli which might influence their knowledge, skills, attitudes, intentions and behavior as well. Nevertheless, comparison with the controls justifies the assumption that the course did have positive effects. Although this study showed that it is feasible to measure positive effects concerning incident reporting after patient safety education, incident reporting is just one of the issues relevant for improving patient safety.

### Future research

Thus, we found that knowledge, attitudes, intentions, and the presence of skills concerning incident reporting, were not sufficient to activate reporting behavior of residents who did not have previous incident reporting experience. To stimulate reporting behavior of these residents, it is necessary to identify and overcome the barriers that are perceived and that discourage the reporting of incidents. To maintain the positive effects on the behavior of residents that were already reporting incidents, another important step is to develop methods to anchor patient safety education throughout the educational career. Assessing the effectiveness of teaching other patient safety issues and other health professionals would also be interesting for future research.

## Conclusions

Patient safety education should be integrated into medical education, as this study showed that patient safety education can have positive effects, both immediately and in the longer-term, on knowledge, skills and attitudes and can modestly influence residents' behavior with respect to reporting incidents. There is, however, a gap between residents' intentions to report incidents and their actual behavior. Therefore, further steps are required to stimulate changes of reporting behavior.

## Competing interests

The authors declare that they have no competing interests.

## Authors' contributions

JDJ collected and analyzed the data, and wrote the manuscript. CW and ABB drew up the educational approach, designed the study and co-wrote the manuscript. All authors read and approved the final manuscript.

## Authors' information

Dr. José D. Jansma carried out this research within the research program Patient Safety in the Netherlands. During this research she was working for the Foreest Medical School of the Medical Center Alkmaar, the Netherlands, and the Department of Public and Occupational Health, EMGO Institute, VU University Medical Center, the Netherlands.

Cordula Wagner, MA, PhD, is professor of patient safety and head of the research area 'Quality and Organization of hospital- and long-term care' at NIVEL Netherlands Institute for Health Services Research. In addition, she is supervisor of the Dutch research program on patient safety in hospitals at the department of public and occupational health/EMGO Institute, VU Medical Center, the Netherlands.

Arnold B. Bijnen, MD, PhD, is a general gastro-intestinal surgeon and worked at the Medical Center Alkmaar. He has been appointed professor of surgery at the VU University Medical Center for teaching and postgraduate training.

## Pre-publication history

The pre-publication history for this paper can be accessed here:

http://www.biomedcentral.com/1472-6963/11/335/prepub

## Supplementary Material

Additional file 1 **Items in questionnaire**. Click here for file
