# Supplementary material for: Effects on incident reporting after educating residents in patient safety: a controlled study
Source: BMC Health Serv Res. 2011 Dec 12;11:335. doi: 10.1186/1472-6963-11-335 (PMC3273445; doi:10.1186/1472-6963-11-335)
Supplement: Additional file 1 — Items in questionnaire. [file 1472-6963-11-335-S1.DOC]

**Additional File.** Items in questionnaire

| 1. I have the feeling that currently I know what to do in case I will be involved in an incident. | |
| --- | --- |
| 2. I have the feeling that currently I am having sufficient knowledge to improve patient safety at my department. | |
| 3. Because of the course I am more able to signal unsafe situations. | |
| 4. Because of the course I can recognize that multiple factors contribute to an incident. | |
| 5. During the course I learned how to analyze incidents systematically. | |
| *Do you consider the following events worth a report?* | |
|  | 6. You bring the wrong patient to the operating room, you notice your mistake in time and pick up the right person. |
|  | 7. At the start of your shift you notice that Mr. B’s heparin pump is adjusted too high. |
|  | 8. You requested with high speed the results of a laboratorial test but you received them much too late. |
|  | 9. The treatment policy of Mrs. X changed, but so far there is no notification of this in her status. |
|  | 10. You notice that the ampoules are not placed as usual, you were not informed about a change in policy. |
|  | 11. On hindsight it became clear that the diagnosis of Mr. M was wrong, the patient did not experience any disadvantages. |
| 12. Do you think it is important for residents to report medical incidents *without* harm for the patient(s)? | |
| 13. Do you think it is important for residents to report medical incidents *with* harm for the patient(s)? | |
| 14. Are you seriously considering reporting medical incidents within the next six months? | |
| 15. Are you planning to start reporting within the next month? | |
| 16. Have you reported a medical incident within the last six months? | |
| 17. If you have, how many incidents did you report within the last six months? | |

Item 1&2: 0=Strongly disagree; 2=Strongly agree. Item 3-16: 0=No; 2=Yes. Item 17. Open question.
